# The *Porphyromonas gingivalis*/Host Interactome Shows Enrichment in GWASdb Genes Related to Alzheimer's Disease, Diabetes and Cardiovascular Diseases

**DOI:** 10.3389/fnagi.2017.00408

**Published:** 2017-12-12

**Authors:** Chris J. Carter, James France, StJohn Crean, Sim K. Singhrao

**Affiliations:** ^1^Polygenic Pathways, Hastings, United Kingdom; ^2^Dementia and Neurodegenerative Diseases Research Group, Faculty of Clinical and Biomedical Sciences, School of Dentistry, University of Central Lancashire, Preston, United Kingdom

**Keywords:** Alzheimer's disease, cardiovascular, diabetes, interactome, *Porphyromonas gingivalis*

## Abstract

Periodontal disease is of established etiology in which polymicrobial synergistic ecology has become dysbiotic under the influence of *Porphyromonas gingivalis*. Following breakdown of the host's protective oral tissue barriers, *P. gingivalis* migrates to developing inflammatory pathologies that associate with Alzheimer's disease (AD). Periodontal disease is a risk factor for cardiovascular disorders (CVD), type II diabetes mellitus (T2DM), AD and other chronic diseases, whilst T2DM exacerbates periodontitis. This study analyzed the relationship between the *P. gingivalis*/host interactome and the genes identified in genome-wide association studies (GWAS) for the aforementioned conditions using data from GWASdb (*P* < 1E-03) and, in some cases, from the NCBI/EBI GWAS database (*P* < 1E-05). Gene expression data from periodontitis or *P. gingivalis* microarray was compared to microarray datasets from the AD hippocampus and/or from carotid artery plaques. The results demonstrated that the host genes of the *P. gingivalis* interactome were significantly enriched in genes deposited in GWASdb genes related to cognitive disorders, AD and dementia, and its co-morbid conditions T2DM, obesity, and CVD. The *P. gingivalis*/host interactome was also enriched in GWAS genes from the more stringent NCBI-EBI database for AD, atherosclerosis and T2DM. The misregulated genes in periodontitis tissue or *P. gingivalis* infected macrophages also matched those in the AD hippocampus or atherosclerotic plaques. Together, these data suggest important gene/environment interactions between *P. gingivalis* and susceptibility genes or gene expression changes in conditions where periodontal disease is a contributory factor.

## Introduction

Complex chronic diseases such as periodontitis, cardiovascular disease (CVD; including atherosclerosis, strokes, hypertension, myocardial infarction, congestive heart failure), type 2 diabetes mellitus (T2DM) and Alzheimer's disease (AD), are increasingly common during advanced aging and hence place a considerable social and economic burden globally. *Porphyromonas gingivalis* is a risk factor for periodontitis and is associated with distal inflammatory pathologies including CVD, T2DM, and AD via immune modification mechanisms. The prevalence of both periodontitis and AD increases with aging (Silvestre et al., 2017) as do CVD (Qiu and Fratiglioni, 2015) and T2DM (Yakaryilmaz and Ozturk, 2017). The aging process itself may contribute to these conditions, particularly in relation to pathogens, via immunosenescence. This can decrease resistance to pathogens due to immunodeficiency but is also accompanied by an increase in the pro-inflammatory activity of monocytes and macrophages which can lead to chronic low grade, inflammation, termed “inflammageing,” which is also associated with AD, CVD, and T2DM (Fülöp et al., 2016).

Periodontitis is of particular interest because of its known polymicrobial etiology and a recognized oral microbiome (Socransky et al., 1998; Aas et al., 2005). The Human Oral Microbiome Database www.homd.org (Chen et al., 2010). An uncontrolled oral microbiome may act as a reservoir, from which opportunistic pathogens can migrate to remote body organs. *P. gingivalis* and oral spirochetes for example, associate with extra-oral niches (Riviere et al., 2002; Poole et al., 2013; Olsen and Progulske-Fox, 2015). Periodontal pathogens enter the systemic system through daily bacteraemia following breakdown of epithelial: endothelial barriers due to the host's inflammatory responses and the ability of some pathogens, including *P. gingivalis* to attack these barriers (Katz et al., 2000).

Chronic periodontitis is associated with the sporadic form of AD and other related comorbidities, T2DM, and atherosclerosis *inter alia* (Löe, 1981, 1993; Olsen and Singhrao, 2015; Singhrao et al., 2015; Olsen et al., 2016; Bale et al., 2017; Harding et al., 2017). Here our focus is on *P. gingivalis* as the keystone pathogen, because it is by far the best-researched bacterium for its contribution to periodontitis (Hajishengallis et al., 2012; Hajishengallis and Lamont, 2014; Olsen et al., 2016).

Periodontal disease is an inflammatory condition affecting the tissues supporting teeth in their bony socket and occurs in aggressive and chronic subtypes. The disease is caused by polymicrobial dysbiosis with several bacterial species playing a significant role in tooth loss (Hajishengallis and Lamont, 2014; Olsen et al., 2016). Some of these include *Actinomycetem actinomycetemcomitans*, and those belonging to the red complex (*P. gingivalis, Tannerella Forsythia, Treponema denticola*). Others are intermediate colonizers of the sub-gingival biofilm ecology of the orange complex, (*Fusobacterium nucleatum, Peptostreptococcus micros, Prevotella intermedia, Prevotella nigrecens, Eubacterium nodatum*, and *Streptococcus constellates*) (Haffajee et al., 2008) and other species. Together they contribute to pathological periodontal pocket formation around the tooth.

The importance of *P. gingivalis* is the bacterium's ability to subvert the roles of organ specific inflammatory cells *via* a number of virulence factors, the most important being its lipopolysaccharide (LPS) and gingipains (Singhrao et al., 2015; How et al., 2016). Once in the new niche, *P. gingivalis* induces dysbiosis of local commensals (Harding et al., 2017) and drives immune reactions in favor of inflammatory amplification whilst maintaining chronic disease (Olsen et al., 2017). The inflammatory contribution from periodontitis links to CVD (atherosclerosis), T2DM and AD and other pathologies. The American Heart Association (AHA) declared, after an extensive review of the literature, that periodontal disease was independently associated with arteriosclerotic vascular disease (ASVD) (Bale et al., 2017). Similarly, T2DM has been recognized as a complication of periodontal disease (Löe, 1993) and periodontitis has been proposed as a risk factor for AD (Kamer et al., 2015; Harding et al., 2017; Leira et al., 2017) with support from longitudinal monitoring documented elsewhere (Chen et al., 2017).

AD is associated with impaired cognition and a number of pathological lesions, classically amyloid-beta (Aβ) deposits and hyperphosphorylated neurofibrillary tangles (Goedert et al., 1991). Many pathogens, (e.g., herpes simplex type 1 (HSV-1), *Chlamydia pneumoniae, Borrelia burgdorferi*) (Itzhaki et al., 2016) as well as *P. gingivalis* lipopolysaccharide (Wu et al., 2017) are able to promote Aβ deposition. Aβ has broad-spectrum antimicrobial effects against bacteria, fungi and viruses (Soscia et al., 2010; White et al., 2014; Bourgade et al., 2015; Kumar et al., 2016) and its deposition may result from an innate immune response to the cerebral invasion of pathogens that have been associated with AD.

T2DM is a metabolic disorder characterized by hyperglycemia and insulin resistance. The complications of diabetes result from long-term elevation of blood glucose levels. Diabetes has significant impact on gingival and periodontal tissues due to poor glycaemic control (Löe, 1981). A consequence of hyperglycaemia is that free sugars circulating in the blood give rise to advanced glycation end-products (AGEs), which are a unique inflammatory product. Endothelial cells and monocytes have receptors that bind AGE products. This in turn has an impact on gingival tissue vascular permeability through enhanced breakdown of the periodontium by protease activity of polymicrobial infections (Embery et al., 2000; Sugiyama et al., 2012). T2DM is a well-established risk factor for stroke (Chen et al., 2016) and as a complication of periodontal disease (Löe, 1981). As with AD, numerous (and similar) oral pathogens have been associated in patients with diabetes (Castrillon et al., 2015). The pancreatic amyloid (amylin) accumulates in pancreatic islets in T2DM. Amylin also has antimicrobial effects and its accumulation may result from an innate immune response to chronic bacterial infections, ultimately triggering the inflammatory pathology related specifically to T2DM (Miklossy and McGeer, 2016).

Periodontitis is associated with age-related diseases such as atherosclerosis *via* immune processes leading to dyslipidaemia in the vessel walls (Libby et al., 2002; Velsko et al., 2014). These events follow from established periodontitis (Socransky et al., 1998). One feature of the polymicrobial infection is that *P. gingivalis* secretes a peptidyl arginine deiminase enzyme that can modify proteins prevalent in atherosclerotic lesions by citrullination (Janssen et al., 2013; Sokolove et al., 2013; Geraldino-Pardilla et al., 2017). Gearldino-Pardilla et al. reported that higher levels of autoantibodies against citrullinated proteins can target citrullinated histone 2B associated with higher coronary artery calcium scores (amount of calcium in walls of arteries supplying heart muscle) when compared with lower antibody levels, suggesting a potential role of seroreactivity to citrullinated histone in atherosclerosis (Geraldino-Pardilla et al., 2017).

All of the aforementioned diseases have both genetic and environmental components, the latter often related to pathogens, as is the case for AD (see above), atherosclerosis (Sessa et al., 2014), and T2DM (Chakraborty et al., 2017). Previous studies have shown that host genes utilized by oncogenic viruses relate to cancer susceptibility genes (Rozenblatt-Rosen et al., 2012) and several genes related to AD are involved in the life cycles of the many pathogens implicated in this disorder (HSV-1, *C. pneumoniae, Cryptococcus neoformans, B. burgdorferi, Helicobacter pylori*, and *P. gingivalis*) (Carter, 2011). The HSV-1 and the parasite *Toxoplasma Gondii*—host interactomes also overlap with the susceptibility genes of a variety of neurological and psychiatric diseases (Carter, 2013a,b). A further study of 110 viruses has shown host/pathogen and host/host interactome overlaps that are relevant to the genetics of multiple human diseases (Navratil et al., 2011). These results support the notion of important relationships between the genes and proteins used by pathogens and disease susceptibility genes. These gene/environment interactions are likely to affect disease manifestation.

In this study, we have compared the *P. gingivalis*/host interactome with GWAS susceptibility genes involved in AD, T2DM and related metabolic (obesity) syndromes, and atherosclerosis (CVD, strokes). We have also compared the gene expression profiles derived from periodontitis gingival tissue or *P. gingivalis*-treated macrophages with those derived from AD hippocampal tissue or atherosclerotic plaques from clinical samples.

## Methodology

The *P. gingivalis*/host interactome (currently comprised of 3,993 host genes) curated manually, from Pubmed references is available at http://www.polygenicpathways.co.uk/pgingivalis.htm, together with a KEGG pathway analysis of the host arm of this interactome.

Susceptibility genes for a variety of diseases were from two genome-wide association study (GWAS) databases. The first of these (GWASdb, http://jjwanglab.org/gwasdb); contains genes with a *P*-value cut-off of *P* < 1E-10^−3^ (Li et al., 2012). (The file downloaded was from the August 2015 release (download GWASdb SNP-Trait file). The second is the more conservative NHGRI-EBI Catalog (*P* < 1E × 10^−5^) posted at (NHGRI-EBI Catalog, https://www.ebi.ac.uk/gwas/docs) accessed in May 2017 (Macarthur et al., 2017) and this was used for selected diseases derived from the initial GWASdb analysis. The reported genes column represents data from the NHGRI-EBI catalog. For AD only, genes labeled as “Alzheimer's disease,” or “Alzheimer's disease (late onset),” were used. GWASdb contains data from 591 disease entities classified as disease ontology identifier (DOIDs) according to Disease-Ontology Lite (http://disease-ontology.org/) (Kibbe et al., 2015). However, many of the keywords used for the searches were general (for example, brain disease, cardiovascular system disease) and these were ignored in favor of more specific identities of interest (e.g., Alzheimer's disease, atherosclerosis, myocardial infarction), (*N* = 285). In addition, we compared the *P. gingivalis* interactome with the proteome of mouse cerebral arteries from the circle of Willis (6,630 proteins) (Badhwar et al., 2014). The proteins from this area of the anatomy is relevant because arteries supplying blood to the brain branch out at the circle of Willis and are subject to atherosclerosis in AD (Roher et al., 2003).

Next, we compared the microarray gene expression studies for periodontitis or *P. gingivalis* with the microarray datasets from AD and/or with atherosclerosis. The periodontitis microarray data are from an integrated analysis of three microarray datasets from human gingival tissue obtained from periodontitis patients (Guo et al., 2015). The effects of *P. gingivalis* relate to a microarray study of human macrophages exposed to live *P. gingivalis*, its LPS, or its fimbrial component (fimbrillin = FimA), 2h post infection or treatment; (Affymetrix Human Genome U133 Plus 2.0 array) (Zhou and Amar, 2007). These effects (periodontitis or *P.gingivalis* microarrays), were compared with microarray datasets obtained from post-mortem studies of the AD hippocampus for incipient and/or established AD (nine control and 22 AD subjects of varying severity on 31 separate microarrays, Affymetrix Human Genome U133A) (Blalock et al., 2004); and from a study comparing stable vs. unstable atherosclerotic plaques derived from human carotid endarterectomy specimens (Total RNA from 11 segments from 3 atherosclerotic plaques classified as stable and unstable: Affymetrix Human Genome U133). The expression data refer to stable/unstable atherosclerotic plaques comparisons (Papaspyridonos et al., 2006). Gene symbols for all data conform to the Human gene Nomenclature (HUGO) system (Povey et al., 2001). Overlapping gene symbols from the comparisons were with the Venny tool, online at the following URL (http://bioinfogp.cnb.csic.es/tools/venny/) (Oliveros, 2007).

### Statistical analysis

Assuming a human genome of 26,846 coding genes and a *P. gingivalis* interactome of 3,993 host genes, 3,993/26,846 gene sets would be expected in any comparator GWAS or other dataset (14.87%). This calculation allowed the determination of the expected values and the enrichment values (observed/expected) in relation to the GWASdb or NHGRI-EBI datasets. The same approach was used to compare microarray data, using the number of misregulated disease genes (= N) to define expected percentage overlap values (= N/26846%). Statistical significance of the enrichment was calculated using the hypergeometric probability test where a Bonferroni cut-off (0.05 × 591 = < 0.0000846) was applied. The resultant *p*-values from each of the analyzed series, was corrected for, by accounting for the false discovery rate (FDR p = q) (Benjamini and Hochberg, 1995). Significant FDR corrected values are considered at *q* < 0.05. The KEGG Pathway enrichment analysis was determined using the Consensus Path database (CPDB) at http://cpdb.molgen.mpg.de/ (Herwig et al., 2016).

## Results

The *P. gingivalis* interactome was highly enriched in GWASdb genes related to neurological disorders (cognitive disorder, dementia and AD), and with metabolic disorders (T2DM and obesity). Each of these conditions has been associated with periodontal disease, as referenced in Table 1. The analysis showed *P. gingivalis* bacterial interactome enrichment in GWASdb genes for CVD including those for hypertension, diverse atherosclerotic conditions, myocardial infarction and congestive heart failure. Similarly, the interactome also related to GWASdb genes from several psychiatric conditions or substance abuse-related disorders that link to periodontal disease (as referenced in Table 1); and are related to mood, depression, anxiety and sleep disorders as well as to substance dependence, including nicotine and alcohol dependency (Table 1). Such a finding was further strengthened by interactome enrichment confirmation in relation to the more stringent NHGRI-EBI GWAS database (*P* < 1E-05) for genes related to AD, T2DM, and atherosclerosis (= carotid plaque burden in the NHGRI-EBI GWAS database). Other diseases were not analyzed.

**Table 1:** The overlaps between the host genes of the *P. gingivalis* interactome with genes associated with diverse diseases from the GWASdb (top unshaded region) or the NCBI-EBI (bottom shaded region) databases.

**Condition**	**Association references**	**N GWAS**	**Overlap**	**Expected**	**O/E**	**Hypergeometric *P*-value**	**FDRp**
**Periodontal**							
Periodontitis: DOID:824 or periodontal disease DOID:3388 (same genes)	Periodontal pathogens include *A. actinomycetemcomitans*, (*P. gingivalis, T. forsythia, T. denticola*) others (*F. nucleatum, P micros, P. intermedia, P. nigrecens, E. nodatum*, and *S. constellates*) (Socransky et al., 1998)	51	11	7	1.47	0.059	NA
**Neurological**							
Cognitive disorder: DOID:1561	Periodontitis has been associated with cognitive decline in middle-aged and older adults (Noble et al., 2009; Naorungroj et al., 2013; Shin et al., 2016) and in Alzheimer's disease (AD) (Ide et al., 2016)	4,763	1,065	698	1.53	1.98E-53	8.36E-52
Dementia: DOID:1307	See AD: Among those aged 75years or older, patients with AD or other types of dementias are at increased risk of poor oral health and poor oral hygiene (Syrjälä et al., 2012). Vascular dementia patients have higher number of decayed teeth and deeper periodontal pockets (Bramanti et al., 2015)	1,645	400	241	1.66	7.96E-26	1.34E-24
Alzheimer's disease: DOID:10652	Periodontitis has been associated with AD (Noble et al., 2009; Sparks Stein et al., 2012; Singhrao et al., 2015) and *P. gingivalis* lipopolysaccharide detected in AD brains (Poole et al., 2013)	1,591	385	233	1.65	1.84E-24	2.86E-23
**Metabolic**							
Type 2 diabetes mellitus: DOID:9352	Periodontitis is associated with type 2 diabetes and higher colonization levels of several periodontal pathogens, including *P. gingivalis* are associated with higher prediabetes prevalence among diabetes-free adults (Demmer et al., 2015). In mice, diabetes increases the risk for periodontal disease induced by *P. gingivalis* but infection did not affect the onset or severity of diabetes in either type 1 or 2 diabetes mice (Li et al., 2013)	3,381	817	495	1.65	2.25E-53	8.86E-52
Obesity: DOID:9970	Periodontal pathogens, including *P. gingivalis* are prevalent in the mouth and stomach of obese individuals undergoing bariatric surgery (Pataro et al., 2016). Bacteria, including *P. gingivalis* associated with body mass index and waist circumference in Japanese subjects (Matsushita et al., 2015)	2,076	501	304	1.65	1.6E-31	3.49E-30
**Cardiovascular**							
Hypertension: DOID:10763	Hypertension and atherosclerosis have been associated with antibodies to *P. gingivalis* (Hanaoka et al., 2013)	1,883	496	276	1.80	1.36E-41	3.48E-40
Arteriosclerosis: DOID:2349	Periodontal bacteria including *A. actinomycetemcomitans, P. gingivalis, T. forsythia, T. denticola* or *F. nucleatum* are believed to contribute to atherosclerosis via effects on lipoprotein serum concentration, endothelial permeability and binding of lipoproteins in the arterial intima (Bale et al., 2017). *P. gingivalis* is the most abundant of over 200 bacterial species detected in non-atherosclerotic coronary and femoral arteries (Mougeot et al., 2017)	823	207	121	1.72	1.73E-15	1.96E-14
Arteriosclerotic cardiovascular disease: DOID:2348		514	130	75	1.73	1.68E-10	1.34E-09
Atherosclerosis: DOID:1936		500	126	73	1.72	3.97E-10	3.08E-09
Myocardial infarction: DOID:5844	A Danish register study (17,691 periodontitis patients) has shown association with myocardial infarction, ischemic stroke, cardiovascular death, and major adverse cardiovascular events (Hansen et al., 2016)	705	180	103	1.74	2.71E-14	2.71E-13
Congestive heart failure: DOID:6000	Periodontitis has been associated with heart failure (Wood and Johnson, 2004; Fröhlich et al., 2016; Holmlund et al., 2017). No specific reports for *P. gingivalis*	233	71	34	2.08	6.27E-10	4.80E-09
**Psychiatric**							
Mood disorder: DOID:3324	See depression	2,536	582	372	1.57	1.84E-30	3.87E-29
Substance dependence: DOID:9973	See alcohol and nicotine	1,518	380	223	1.71	4.57E-27	7.92E-26
Alcohol dependence: DOID:0050741	High levels of periodontal pathogens, including *P. gingivalis* observed in alcoholic patients (Amaral et al., 2011; Sender-Janeczek and Zietek, 2016). Alcohol consumption is a risk factor for periodontitis (Shepherd, 2011)	1,139	283	167	1.69	6.93E-20	9.08E-19
Nicotine dependence: DOID:0050742	Low concentrations of cigarette smoke condensate increase invasion of human gingival epithelial cells by *P. gingivalis* (Imamura et al., 2015)	542	149	79	1.87	8.26E-15	8.71E-14
Anxiety disorder: DOID:2030	Social stress enhances the inflammatory response to *P. gingivalis* in mice (Bailey et al., 2009)	360	91	53	1.7	7.38E-08	4.35E-07
Sleep disorder: DOID:535	Periodontal disease has been associated with obstructive sleep apnoea (meta-analysis) (Al Jewair et al., 2015). Sleep deprivation increases periodontitis in a rat model (Nakada et al., 2015). No reports for *P. gingivalis*.	247	67	36	1.85	2.31E-07	1.22E-06
Endogenous depression: DOID:1595/major depressive disorder: DOID:1470 (same genes)	The incidence of depression is higher in patients with periodontitis (Hsu et al., 2015; Kumar et al., 2015). No specific links found for *P. gingivalis*	510	109	75	1.46	1.73E-05	6.99E-05
**Blood disorders**							
Leukopenia: DOID:615	See agranulocytosis	995	225	146	1.54	1.08E-11	9.27E-11
Agranulocytosis: DOID:12987	= Neutropenia: Neutropenia has been associated with prepubertal periodontitis and with the subgingival microflora, including *P. gingivalis* (Kamma et al., 1998)	376	99	55	1.80	2.44E-09	1.73E-08
**NCBI-EBI GWAS**							
Alzheimer's disease		78	32	11.4	2.8	1.47E-08	5.90e-08
Type 2 diabetes		217	58	31.8	1.82	2.18E-06	4.35E-06
Atherosclerosis carotid plaque burden)		46	14	6.7	2.08	0.0035	0.0047
Periodontitis		108	22	15.8	1.39	0.029	0.029

*The total number of associated GWAS genes (N GWAS) is shown for each disease, together with the number of these genes common to the P. gingivalis interactome (overlap). The expected number of common genes and the observed/expected ratios (O/E) are also shown together with the p-value for enrichment and the P-value corrected for false discovery (FDRp). NS, non-significant (see section Methodology)*.

*DOID, Disease Ontology identifier; O/E, ratio of overlapping genes to expected genes; O, genes found to overlap with P. gingivalis interactome; E, number of genes expected to overlap by random chance; FDRq, p (q)-value corrected for False discovery*.

The overlap between the *P. gingivalis* interactome and periodontitis/periodontal disease (GWASdb) was not significant for 51 GWASdb genes, but was nominally significant (*p* = 0.029) for 108 periodontitis genes from the NHGRI-EBI Catalog. This lack of, or low significance may relate to the fact that numerous other pathogens are involved in causing chronic periodontitis (Table 1). It could also be related to statistical power problems inherent to this type of analysis (a fixed number of the *P. gingivalis* interactome (*N* = 3993) and variable numbers of GWASdb susceptibility genes (from 51 in periodontitis to 4,763 in cognitive disorder).

### A focus on AD, T2DM, atherosclerosis and hypertension

**Figure 1:**
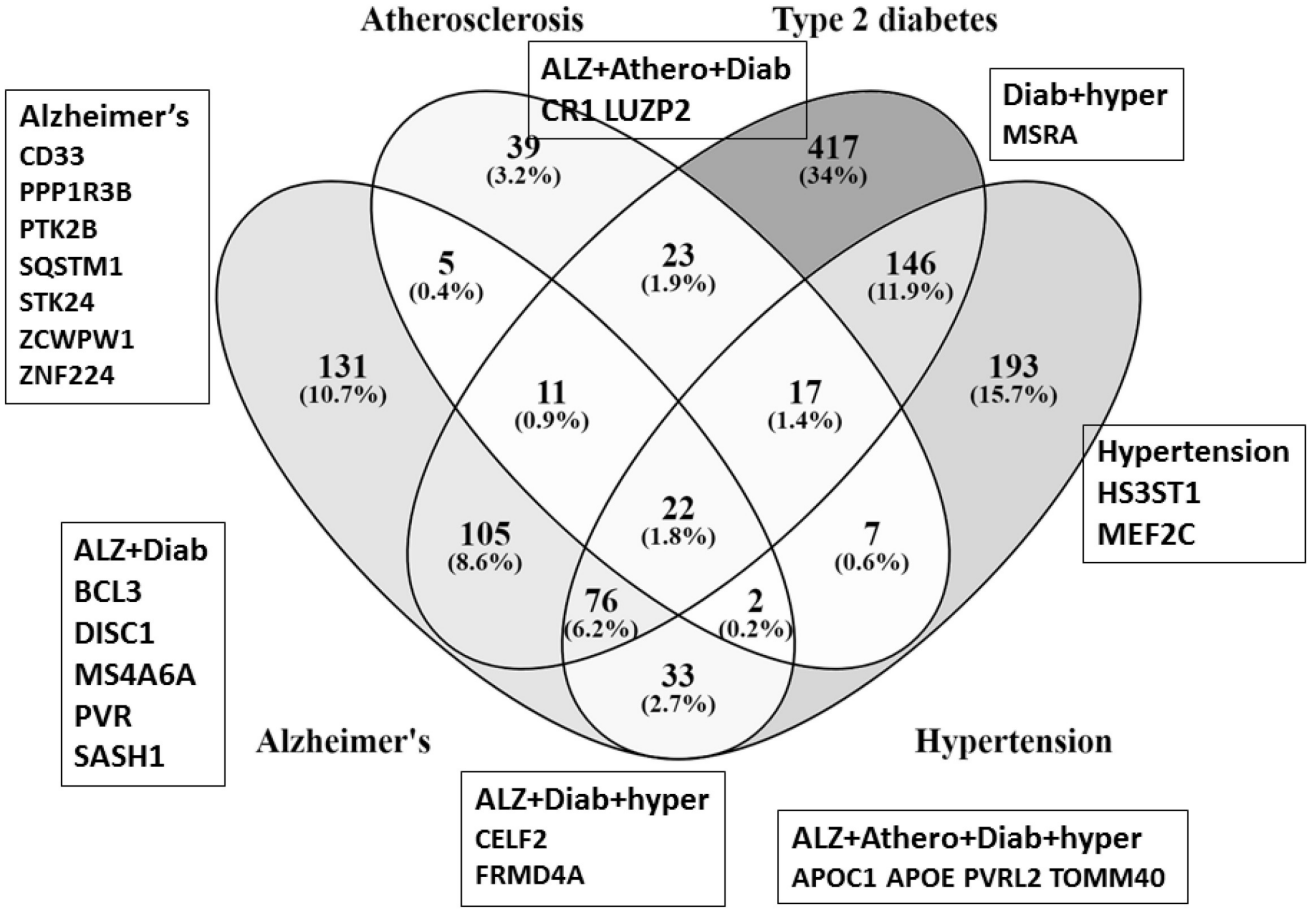
Venn diagram shows the number of susceptibility genes (GWASdb) and their percentage occurrence within the *P. gingivalis*/host interactome that are common or specific to various combinations of atherosclerosis (Athero), type 2 diabetes (Diab), hypertension (hyper) and Alzheimer's (Alz), The Venn diagram is for data from GWASdb. The genes in boxes refer to GWAS genes from the more stringent NCBI-EBI data that are also part of the *P. gingivalis*/host interactome. The percentages are those of the sum of the interactome/susceptibility gene overlaps for all diseases represented in the figure (atherosclerosis+ type 2 diabetes + hypertension+ Alzheimer's gene/interactome overlaps).

The *P. gingivalis* interactome overlaps with GWASdb genes associated with each of these (AD, T2DM, atherosclerosis and hypertension) diseases or risk factors. Some of these interactome overlaps are with genes specific to either single diseases or risk factors (diabetes = *N* = 417; hypertension *N* = 193; AD = *N* = 131; atherosclerosis *N* = 39). In other cases, overlapping interactome genes are common to two or more diseases such as, AD/diabetes *N* = 105; AD/atherosclerosis *N* = 22; AD/atherosclerosis/diabetes *N* = 11; (AD/atherosclerosis/hypertension *N* = 5) (Figure 1). Twenty-three of seventy-eight AD genes from the more stringent NHGRI–EBI GWAS database (*p* < 1E-05) also appeared in these four datasets (Figure 1).

### Over-representation of arterial genes in the *P. gingivalis* interactome

Of the full *P. gingivalis* interactome (3,993 genes), 1,423 belonged to the cerebral artery proteome dataset of 6,630 proteins (21.4%: Observed/expected = 1.44: enrichment: *P* = 7.2E-63). Of the 385 genes common to the *P. gingivalis* interactome and AD GWASdb genes, 175 belonged to the cerebral artery proteome: (45.3%; observed/expected = 1.85: enrichment *P* = 0).

### KEGG pathway analysis of the AD genes common to the *P. gingivalis* interactome

The analysis of pathways outlined by the GWASdb genes common to AD and the *P. gingivalis* interactome generated by the CPDB website, are in Table 2. (KEGG pathways for the entire host/pathogen interactome are posted at http://www.polygenicpathways.co.uk/pgingkegg.html. These genes include pathways related to cancers, AD, infection and immune responses, cytokine and chemokines and multiple metabolic signaling processes). KEGG pathway analysis of the common AD/interactome genes revealed pathways relevant to blood-brain barrier (BBB) function, including focal adhesion, junction and actin pathways, protein digestion and absorption (mainly represented by collagen genes that are related to vascular smooth muscle, and leukocyte trans endothelial migration).

**Table 2:** KEGG pathway enrichment analysis of the genes common to GWASdb AD genes and the *P. gingivalis* interactome.

**KEGG pathway**	***p*-value**	***q*-value**	**Overlapping genes**
**BARRIER RELATED**			
Focal adhesion	0.0001	0.006	ACTN1; PTK2; PDGFD; PRKCA; ITGA1; ITGA4; MYLK; AKT3; MAPK10; COL4A2; COL4A1; ACTB; BCAR1; EGF
Protein digestion and absorption	0.0002	0.006	COL18A1; COL14A1; COL5A2; SLC8A1; COL27A1; COL4A2; COL4A1; MME; DPP4
Vascular smooth muscle contraction	0.001	0.019	GNAS; PRKCA; PRKCE; RAMP1; MYLK; KCNMA1; PRKG1; ITPR3; CALD1
Leukocyte transendothelial migration	0.004	0.03	ACTN1; PTK2; PRKCA; ITGA4; RASSF5; PTK2B; ACTB; BCAR1
Adherens junction	0.005	0.03	TGFBR2; ACTN1; SMAD3; PTPRB; PTPN1; ACTB
Gap junction	0.013	0.07	PDGFD; GNAS; PRKCA; PRKG1; ITPR3; EGF
Regulation of actin cytoskeleton	0.02	0.08	ACTN1; PTK2; PDGFD; CYFIP2; ITGA1; ITGA4; MYLK; ACTB; BCAR1; EGF
**DISEASES**			
Arrhythmogenic right ventricular cardiomyopathy	0.0001	0.006	ACTN1; ITGA1; CACNB1; SGCD; ITGA4; SLC8A1; LMNA; ACTB
Pathways in cancer	0.0002	0.006	TGFBR2; NOS2; PTK2; AXIN1; GNAS; MECOM; RASSF5; SMAD3; CSF1R; CYCS; PRKCA; GLI2; PPARG; AKT3; GNG2; COL4A2; COL4A1; GNB4; MAPK10; EPAS1; EGF
Dilated cardiomyopathy	0.0008	0.013	GNAS; ITGA1; CACNB1; SGCD; ITGA4; SLC8A1; ACTB; LMNA
Hypertrophic cardiomyopathy	0.002	0.023	ITGA1; CACNB1; SGCD; ITGA4; SLC8A1; LMNA; ACTB
Colorectal cancer	0.002	0.023	TGFBR2; AXIN1; CYCS; SMAD3; AKT3; MAPK10
Non-small cell lung cancer	0.008	0.04	PRKCA; FOXO3; AKT3; RASSF5; EGF
Small cell lung cancer	0.011	0.06	NOS2; PTK2; CYCS; AKT3; COL4A2; COL4A1
Pancreatic cancer	0.015	0.07	TGFBR2; AKT3; MAPK10; EGF; SMAD3
Endometrial cancer	0.03	0.09	FOXO3; AKT3; EGF; AXIN1
Insulin resistance	0.03	0.1	PPP1R3B; PRKCE; AKT3; MAPK10; PTPN1; CREB3L1
Alzheimer's disease	0.04	0.11	APOE; ATP2A3; CYCS; GRIN2A; MAPT; SNCA; MME; ITPR3
Transcriptional misregulation in cancer	0.045	0.12	TGFBR2; PTK2; CSF1R; HMGA2; AFF1; CD86; PPARG; MEIS1
**IMMUNE**			
AGE-RAGE signaling pathway in diabetic complications	0.0003	0.008	TGFBR2; PRKCA; PRKCE; SMAD3; AKT3; JAK2; COL4A2; COL4A1; MAPK10
Hematopoietic cell lineage	0.005	0.033	CR1; CSF1R; ITGA1; CD33; ITGA4; IL6R; MME
Inflammatory mediator regulation of TRP channels	0.02	0.09	GNAS; CAMK2D; PRKCE; PRKCA; MAPK10; ITPR3
Th17 cell differentiation	0.03	0.1	STAT6; TGFBR2; SMAD3; JAK2; MAPK10; IL6R
**SIGNALING**			
Calcium signaling pathway	0.0002	0.006	NOS2; GNAS; CAMK2D; ATP2A3; MYLK; CACNA1G; PRKCA; ADRB2; PTK2B; SLC8A1; ITPR3; ATP2B2; GRIN2A
PI3K-Akt signaling pathway	0.0002	0.006	PTK2; PDGFD; OSMR; GHR; CSF1R; PRKCA; ITGA1; EFNA5; ITGA4; IL6R; FOXO3; AKT3; JAK2; COL4A2; COL4A1; GNB4; GNG2; EGF; CREB3L1
Circadian entrainment	0.0003	0.007	GNAS; CAMK2D; GNB4; PRKG1; CACNA1G; PRKCA; GRIN2A; GNG2; ITPR3
cGMP-PKG signaling pathway	0.001	0.019	ATP2A3; PRKCE; MYLK; KCNMA1; ADRB2; AKT3; PRKG1; SLC8A1; ITPR3; ATP2B2; CREB3L1
Rap1 signaling pathway	0.003	0.024	PDGFD; GNAS; PRKCA; RASSF5; EFNA5; CSF1R; AKT3; ACTB; SIPA1L2; BCAR1; EGF; GRIN2A
ErbB signaling pathway	0.003	0.025	PTK2; NCK2; CAMK2D; PRKCA; AKT3; MAPK10; EGF
Ras signaling pathway	0.005	0.032	PDGFD; PRKCA; CSF1R; EFNA5; RASSF5; PLA1A; AKT3; MAPK10; GNB4; GNG2; EGF; GRIN2A
Adrenergic signaling in cardiomyocytes	0.006	0.033	GNAS; CAMK2D; CACNB1; PRKCA; ADRB2; AKT3; SLC8A1; ATP2B2; CREB3L1
Phospholipase D signaling pathway	0.014	0.07	PDGFD; GNAS; PRKCA; EGF; PTK2B; AKT3; GRM8; DGKI
GnRH signaling pathway	0.016	0.07	GNAS; CAMK2D; PRKCA; PTK2B; MAPK10; ITPR3
Hippo signaling pathway	0.02	0.08	TGFBR2; AXIN1; WWC1; SMAD3; GLI2; ACTB; FRMD6; TEAD4
MAPK signaling pathway	0.03	0.09	TGFBR2; MECOM; CACNB1; CACNA1G; PRKCA; AKT3; MAPK10; MAPT; RELB; DUSP16; EGF
HIF-1 signaling pathway	0.03	0.09	NOS2; CAMK2D; IL6R; PRKCA; AKT3; EGF
cAMP signaling pathway	0.03	0.1	GNAS; CAMK2D; CREB3L1; ADRB2; AKT3; MAPK10; ABCC4; ATP2B2; GRIN2A
**NEURAL**			
Dopaminergic synapse	0.0006	0.012	GNAS; CAMK2D; CREB3L1; PRKCA; AKT3; GNG2; MAPK10; GNB4; ITPR3; GRIN2A
Axon guidance	0.002	0.023	SEMA3A; PTK2; NGEF; SEMA5A; NCK2; CAMK2D; EFNA5; PRKCA; ABLIM1; EPHA5; SEMA5B
Cholinergic synapse	0.003	0.025	CAMK2D; GNB4; PRKCA; GNG2; AKT3; JAK2; ITPR3; CREB3L1
Glutamatergic synapse	0.004	0.027	GRM8; GNAS; PRKCA; GNB4; GRIK2; GRIN2A; GNG2; ITPR3
Amphetamine addiction	0.02	0.07	CAMK2D; PRKCA; GRIN2A; CREB3L1; GNAS
Long-term depression	0.04	0.12	PRKCA; ITPR3; PRKG1; GNAS
**HORMONES/SECRETION**			
Gastric acid secretion	0.001	0.019	GNAS; CAMK2D; MYLK; KCNK2; PRKCA; ITPR3; ACTB
Aldosterone synthesis and secretion	0.002	0.024	GNAS; CAMK2D; PRKCE; CACNA1G; PRKCA; CREB3L1; ITPR3
Vasopressin-regulated water reabsorption	0.003	0.024	DYNC1I1; CREB3L1; GNAS; DYNC2H1; DYNC1H1
Salivary secretion	0.004	0.027	GNAS; PRKCA; KCNMA1; ADRB2; PRKG1; ITPR3; ATP2B2
Insulin secretion	0.01	0.06	GNAS; CAMK2D; KCNMA1; PRKCA; CREB3L1; ITPR3
Pancreatic secretion	0.02	0.08	ATP2A3; PRKCA; GNAS; KCNMA1; ITPR3; ATP2B2
**INFECTION**			
Amoebiasis	0.006	0.033	ACTN1; PTK2; GNAS; PRKCA; NOS2; COL4A2; COL4A1
Salmonella infection	0.01	0.06	NOS2; DYNC1I1; MAPK10; DYNC1H1; ACTB; DYNC2H1
Hepatitis B	0.016	0.07	STAT6; PRKCA; CYCS; SMAD3; PTK2B; AKT3; MAPK10; CREB3L1
Tuberculosis	0.018	0.07	NOS2; CR1; CAMK2D; LSP1; CYCS; AKT3; JAK2; MAPK10; PIK3C3
Chagas disease (American trypanosomiasis)	0.028	0.09	TGFBR2; NOS2; GNAS; SMAD3; AKT3; MAPK10
Viral myocarditis	0.039	0.11	ACTB; SGCD; CYCS; CD86
**OTHER**			
Choline metabolism in cancer	0.02	0.09	PDGFD; PRKCA; DGKI; AKT3; MAPK10; EGF
beta-Alanine metabolism	0.03	0.1	ALDH6A1; ALDH1A3; DPYD
Regulation of lipolysis in adipocytes	0.035	0.1	ADRB2; AKT3; PRKG1; GNAS
Osteoclast differentiation	0.0025	0.02	TGFBR2; SQSTM1; CSF1R; PPARG; AKT3; MAPK10; TNFRSF11B; TREM2; RELB
Apoptosis	0.036	0.106164	CASP6; CYCS; AKT3; MAPK10; ITPR3; ACTB; LMNA

*The statistical significance (p-values) and false discovery corrected P-values (q-values) as determined by the CPDB website*.

### Microarray studies of periodontitis or *P. gingivalis*: comparison with alzheimer's disease and atherosclerosis

#### Periodontitis comparison

**Figure 2:**
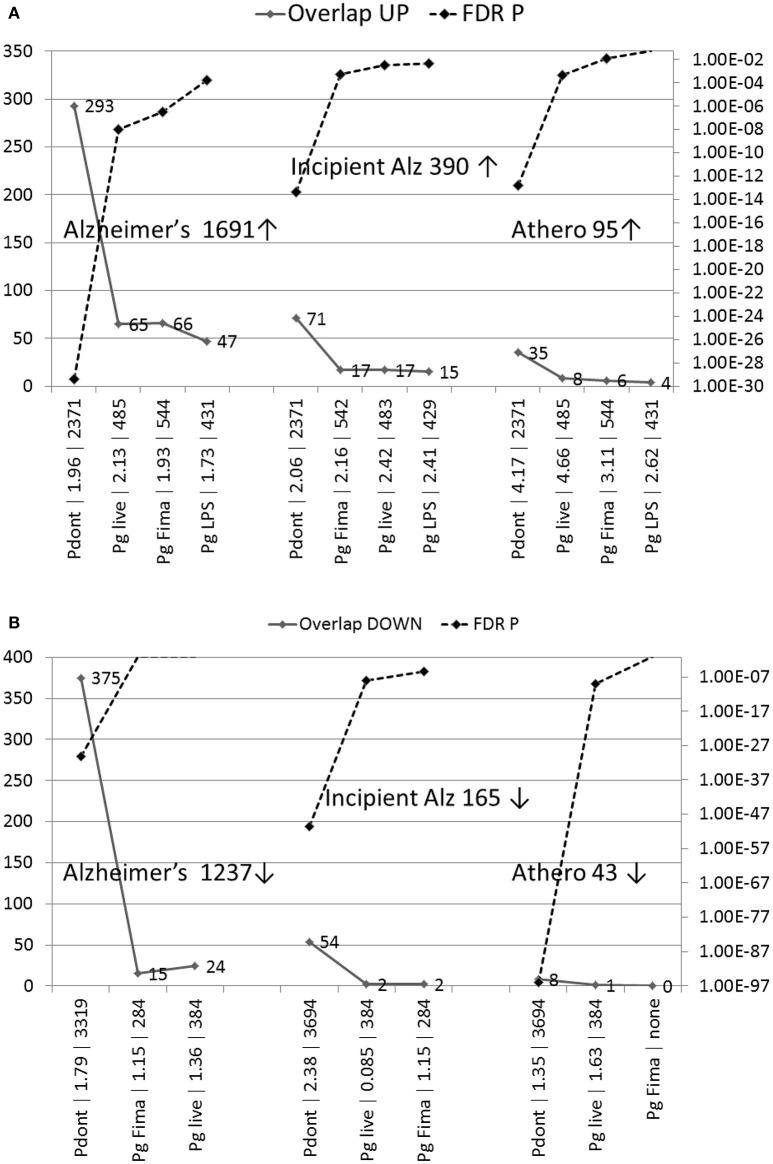
**(A,B)** The number of overlapping genes and the significance of enrichment in comparisons of the effects of periodontitis in human oral tissue or *P. gingivalis* in human macrophages with microarrays from the AD hippocampus or from unstable atherosclerotic plaques (Athero). **(A)** Upregulated genes; **(B)** Down-regulated genes. Pdont, periodontitis; Pg live, live *P. gingivalis*; Pg LPS, *P. gingivalis*-lipopolysaccharide; Pg Fima, *P. gingivalis* fimbrial component. The labels on the X-axis (e.g., Pdont | 1.96 | 2371) correspond to these conditions, followed by observed/expected values, and the number of misregulated genes in the periodontitis or *P. gingivalis* microarrays. The maximum of the Y-axis (FDR *q*-value) is set at *p* = 0.05 and invisible points above this line are non-significant. No down-regulated data were available for down-regulated genes in the case of *P. gingivalis* LPS. The number of up or down-regulated genes in the Alzheimer's (Alz) or atherosclerosis datasets are also shown.

The genes upregulated in periodontitis significantly overlapped with those upregulated in the AD hippocampus [mature AD (*P* = 3.9E-30), incipient AD (*P* = 4.6E-14)] and in atherosclerotic plaques (*P* = 1.7E-13) (Figure 2A). There was also significant match between the genes downregulated in periodontitis and those downregulated in mature AD (*P* = 5.6E-31), incipient AD (*P* = 3.5E-51) and atherosclerotic plaques (*P* = 9.8E-97) (Figure 2B).

#### P. gingivalis comparison

In general, the overlaps between the *P. gingivalis* microarrays (live, LPS, or FimA effects in macrophages) and the misregulated genes in AD or atherosclerosis were fewer and less significant, than when compared with periodontitis, where a more diverse microbial population is encountered (Figures 2A,B). The genes upregulated in AD significantly matched those upregulated by live *P. gingivalis* (mature AD: *P* = 1.1E-08: incipient AD: *P* = 0.0003), its LPS (mature AD: *P* = 0.0002: incipient AD: *P* = 0.005) or FimA (mature AD: *P* = 3.2E-07: incipient AD *P* = 0.0006) (Figure 2A). The genes downregulated in incipient AD matched those affected by live *P. gingivalis* infection (*P* = 1.1E-08) or its FimA (*P* = 3.4E-06) but the comparisons between mature AD and live *P. gingivalis* or its FimA component were not significant (Figure 2B).

The genes upregulated in atherosclerotic plaques also matched those affecting macrophages by live *P. gingivalis* (*P* = 0.0005) infection, or its FimA component (*P* = 0.013). The LPS comparison was not significant (*P* = 0.06). The effects of live *P. gingivalis*, were significant for the genes downregulated in the atherosclerosis comparison (*P* = 9.2E-10). The FimA comparison was not significant (*P* = 0.06) and no downregulated data were available for the bacterial LPS.

## Discussion

*P. gingivalis* is but one of several pathogens contributing to periodontal disease and this may contribute to the lack of (GWASdb) or low significance (NHGRI–EBI) of the overlap between the host/pathogen interactome and the genes associated with periodontitis itself. Many individuals do not develop periodontitis during their lifetime (Trombelli et al., 2004). Progression to the aggressive form of periodontitis is also rare and is associated with neutrophil defects with *A. actinomycetemcomitans* as the dominant etiological agent (Zambon, 1985), whereas chronic periodontitis is associated with complexes containing mixtures of species such as *P. gingivalis, T. forsythia* and a range of spirochetes (Socransky et al., 1998; Haffajee et al., 2008). Given the relationship between pathogens and host genes, as exemplified in this paper, such clinical variables may also depend upon the host's genetic profile. However, the enrichment of the interactome in genes associated with many other diseases linked to periodontitis (e.g., AD, CVD, and T2DM) was highly significant. This suggests that, within the sub-gingival microbiome, *P gingivalis* is a key pathogen contributing to the pathologies of these diseases. The focus of this paper relates principally to AD through these comorbid states.

The oral pathogen *P. gingivalis* interacts with or alters the expression of thousands of host genes forming an extensive host/pathogen interactome. The genes of the host arm of this interactome overlap with GWASdb genes associated with AD, atherosclerosis and T2DM and other CVD complications including hypertension and myocardial infarction. They also overlap with GWASdb genes associated with several psychiatric conditions and/or psychological factors determining life-styles including mood disorders, depression, anxiety and sleep disorders and substance abuse. Periodontitis can affect mood, depression, anxiety and sleep disorders as discussed previously (Harding et al., 2017) and the outcome of this interactome analysis supports this related conclusion. In addition, dependency on drugs including nicotine and excessive alcohol intake can negatively affect oral hygiene and periodontitis (Bonfim et al., 2013; Singh et al., 2013) and subsequently mental health as a bi-directional association (oral health < > mental health) (Kisely, 2016). The overlap between the genes associated with nicotine and alcohol abuse and the *P. gingivalis* interactome (Table 1) may relate to the effects of nicotine or alcohol on host genes affecting pathogen levels and virulence. For example, nicotine reduces the induction of the anti-microbial beta-defensin-2 induced by *P. gingivalis* LPS in human gingival epithelial cells (Mahanonda et al., 2009), and even low concentrations of cigarette smoke condensate increase invasion of human gingival epithelial cells by *P. gingivalis* (Imamura et al., 2015). In general, both alcoholism and smoking can modify cytokine responses to bacterial LPS or lipoteichoic acid in systemic immune cells (Gaydos et al., 2016).

Polymorphisms in human genes affect host physiology, but they are also likely to affect the interactions between pathogen and host. Pathogens also use these host genes/proteins during their life cycles, and the host employs many immune, inflammatory and defensive genes against the pathogens. Modifications in the host genes must also impinge on these effects. As previously discussed (Carter, 2013a,b), disease susceptibility genes relevant to pathogens may thus divert the effects of the pathogen toward or away from particular pathways, thus orientating its adverse effect to directions that enable it to influence diverse diseases in a manner that depends on the genetics of the host. The effects of *P. gingivalis* on colonization, biofilm formation, immune responses and endothelial cell activation are also strain-dependent (Walter et al., 2004; Belanger et al., 2008; Wilensky et al., 2009; Barbosa et al., 2015) and it is likely that distinct host gene relationships and disease relationships exist for strains with differing virulence.

In this study, the host genes of the bacterial interactome coincided with a high degree of significance with susceptibility genes related to cognitive disorders, AD and dementia, as well as with genes related to obesity and T2DM, and to those related to atherosclerosis and related disorders. This study adds significance to periodontal disease or *P. gingivalis* infection having associations with all of these conditions. The *P. gingivalis* interactome related to genes specifically or commonly associated with AD, atherosclerosis, diabetes and hypertension as depicted in the Venn diagram. These conditions are inter-related. For example diabetes (Shinohara and Sato, 2017), atherosclerosis (Hofman et al., 1997), and hypertension (de la Torre, 2006) are all associated with AD, as is obesity (Milionis et al., 2014). *P. gingivalis* may contribute to each condition in diverse ways via these host/pathogen interactions. The pathogen may thus influence AD risk both directly and via its influence on these other AD risk factors. There have been fewer studies relating periodontal disease to psychiatric disorders such as schizophrenia (McCreadie et al., 2004) but this study suggests that any such relationship may also have genetic components.

The relationship between periodontitis or *P. gingivalis* and AD or atherosclerosis is supported by the transcriptome analyses studies. The gene expression signatures in periodontal disease tissue or in macrophage responses to *P. gingivalis* components significantly matched those from the AD hippocampus or from atherosclerotic plaques in most cases, particularly in relation to the upregulated genes. In the case of the AD hippocampal transcriptome the upregulated genes contain the pathways relevant to pathogens and immune activation (inflammation, complement activation, and the defense response) (Blalock et al., 2004). The degree of significance was higher for the periodontal disease transcriptome and this may reflect the contribution of many other pathogens involved in periodontitis. AD (Itzhaki et al., 2016) and atherosclerosis (Sessa et al., 2014; Budzynski et al., 2016) have also been associated with multiple pathogens and recent microbiome studies have reported numerous bacterial and fungal species, some of oral origin, in the AD brain (Emery et al., 2017; Pisa et al., 2017). In relation to AD, the KEGG pathway analysis of the overlap between the interactome/AD genes provides some clues as to how *P. gingivalis* may contribute to AD. Many significantly affected pathways relate to breakdown of functional barriers and to the immune system and inflammatory pathways. The host genes of the bacterial interactome were also highly enriched in those found in the cerebral artery of the circle of Willis.

Periodontitis and atherosclerosis can be induced in apolipoprotein E knockout (ApoE^−/−^) mice (Velsko et al., 2014). We have reported such investigations (Poole et al., 2015; Singhrao et al., 2017). A recent study using the same ApoE^−/−^ *P. gingivalis-*infected mice brains (24 weeks) provided evidence of BBB deterioration, and cerebral tissue damage (Singhrao et al., 2017). The BBB breach may have resulted from acute phase inflammation in the form of oxidative stress and *P. gingivalis* protease (gingipains) activity in these mice (Rokad et al., 2017; Singhrao et al., 2017). Gingipains have been shown to degrade collagens (Bedi and Williams, 1994; Zhou and Windsor, 2006), and cell-cell adhesion molecules (cadherins and integrins) contributing to endothelial and epithelial barrier disruption (Katz et al., 2000; Hintermann et al., 2002; Sheets et al., 2005).

Disruption of the BBB is an early feature of AD (van de Haar et al., 2016), and *P. gingivalis* may be one of the many factors contributing to this adverse outcome. The weakened BBB may enable the cerebral entry of other pathogens (viral, fungal, and bacterial origin), that are detected in AD brains. The leaky BBB might also favor the entry of other environmental toxins (e.g., pesticides, pollution, aluminum, or heavy metals) that have been associated with Aβ fibrils in AD (Carter, 2017). In addition, the *P. gingivalis* interactome was highly enriched in cerebral arterial genes, which further supports the correlation with BBB damage. Although *P. gingivalis* LPS is able to provoke Aβ deposition (Wu et al., 2017), the bacterial interactome genes overlapping with the AD GWASdb genes were not significantly enriched in the KEGG AD pathway, suggesting that the BBB-related effects of *P. gingivalis* may be of greater relevance to its developing pathology. In the periphery, vessel damage likely by gingipains may also allow bacteria to enter the circulatory system and cause transient bacteraemia that contribute to atherosclerosis (Bahrani-Mougeot et al., 2008; Lockhart et al., 2008; Singhrao et al., 2017) and possibly insulin resistance. The endothelial vessel barrier is disrupted in diabetes and in atherosclerosis (Chistiakov et al., 2015; Dong et al., 2016) showing common defects with AD.

There are several general caveats to this type of analysis. For example, the susceptibility gene/interactome overlaps deal with gene symbols rather than with specific polymorphisms, and there have been no studies linking host genetic polymorphisms to specific components of the *P. gingivalis* life cycle. The effects of the pathogen may also be strain-specific and some strains may be more virulent. Within any large interactome, effects in relation to the host may be null, deleterious or even beneficial. The statistical analyses similarly rely on the degree of overlap between two sets of gene symbols, with no indication of physiological weight or direction. However, any of the individual gene effects noted accordingly referenced on the following website (http://www.polygenicpathways.co.uk/pgingivalis.htm). The pathway analyses similarly depend upon those made available by particular websites and pathways that are more relevant may exist but remain inaccessible to the wider public.

## Conclusions

The host genes employed by *P. gingivalis* during its life cycle, and those reacting to infection by the pathogen-host interaction are enriched in GWASdb genes for several diseases where the pathogen is suspected to play a contributory role. These notably include AD, T2DM, and atherosclerosis. This was confirmed for associated genes in the more stringent NHGRI-EBI GWAS database. The genes misregulated in periodontal tissue or by *P. gingivalis* or its components in macrophages also relate to those misregulated in AD and atherosclerosis. It may be plausible to suggest that the major effects of *P. gingivalis* relate to its ability to disrupt barrier function and this could play a key role in its downstream pathological events. Disrupted BBB function caused by *P. gingivalis* may also be relevant to the many other pathogens that have been associated with AD and related diseases.

From a genetic standpoint, it is increasingly clear that disease susceptibility genes relate not only to human physiology, but also to that of the pathogens implicated in the same disease. This suggests that pathogens and genes condition each other's effects. It is not yet clear how or whether polymorphisms in these susceptibility genes influence the disease promoting effects of this pathogen, and, in general, there has been little work in this area. It is also likely that such gene/pathogen interactions could determine the extent to which *P. gingivalis* can influence the acute or chronic aspects of periodontitis or other components of the oral microbiome. From a medical standpoint it is however evident that the prevention of periodontal disease or strategies directed at keystone pathogens such as *P. gingivalis* could have a major effect on the incidence and progression of AD, cardiovascular diseases and type 2 diabetes, and possibly many other disorders.

## Future perspectives

This bioinformatics analysis supports the many documented relationships between *P. gingivalis* infection and AD or its comorbid conditions, T2DM and atherosclerosis. The key effects of the pathogen may relate to barrier disruption and inflammatory processes. Further studies should include animal models to determine whether this pathogen can damage and breach the BBB and whether it can access the brain and promote the hallmark pathologies of AD, including Aβ deposition, tau phosphorylation, inflammation and neuronal death in relevant areas. It is also likely that targeting this and other periodontal pathogens could be of benefit to a variety of human diseases.

## Author contributions

CC: Manually curated the *P. gingivalis*/host interactome, created the figures and tables. CC, JF, and SS contributed to the many draft versions of all sections of the manuscript. SC provided critical feedback and funding.

### Conflict of interest statement

The authors declare that the research was conducted in the absence of any commercial or financial relationships that could be construed as a potential conflict of interest.
